# Nurses' knowledge, perception and practice toward discharge planning in acute care settings: A systematic review

**DOI:** 10.1002/nop2.547

**Published:** 2020-06-24

**Authors:** Audai A. Hayajneh, Issa M. Hweidi, Milian W. Abu Dieh

**Affiliations:** ^1^ Adult Health Nursing Department Faculty of Nursing Jordan University of Science and Technology Irbid Jordan; ^2^ Adult Health Nursing Department School of Nursing Jordan University of Science and Technology Irbid Jordan; ^3^ School of Nursing Jordan University of Science and Technology Irbid Jordan

**Keywords:** acute care, discharge planning, knowledge, nurses, perception, practice, systematic review

## Abstract

**Aim:**

Discharge planning (DP) guides patients' transition to out‐hospital services. This systematic review investigates nurses' knowledge, perception and practices of discharge planning.

**Design:**

We conducted a systematic review following PRISMA guidelines.

**Methods:**

Search terms were used to identify research studies published between 1990–2020 across six databases: CINAHL, MEDLINE, PubMed, Complete Academic search, Science Direct and Google Scholar. A total of nine studies met the inclusion criteria.

**Results:**

Nine articles revealed nurses' knowledge, perspectives and practices of discharge planning. Obstacles included low‐level knowledge of patients' activities and discharge; inability to define DP; debates over the timing of beginning, implementing and preparing discharge; patients and their family members' negative attitudes towards DP; and perceiving DP as excessive, time‐consuming paperwork for which the physician is responsible. Better time management during work improves DP in acute care settings.

## 1 INTRODUCTION

Historically, discharge planning (DP) has been defined as the activities that guide patients to community services after being discharged from any care setting (Abdulfattah & Mushcab, 2017). It was concerned only with the physical aspects of the patients. However, over time, the word “planning” began to involve short‐term goals, such as predicting changes in the patients' needs and long‐term goals, such as providing continuous care. Today, the main aim of DP is to help patients progress through various levels of care. For instance, the needs may need to be met in critical care units or regular wards (Abdulfattah & Mushcab, 2017). DP is now considered a process, not a single event. The process ought to begin at admission and proceed until the patient is allocated to the next level of health care (Birjandi & Bragg, 2008). The patient should receive the understanding, motivation and skills necessary for self‐management at home (Flink & Ekstedt, 2017).

## 2 BACKGROUND

Discharge planning sets the foundation for effective changes in patients as they move from hospitals to their homes (Nordmark, Zingmark, & Lindberg, 2016; Pellett, 2016). It prepares individuals and their families for independent self‐care by providing them with appropriate support and resources in their community. Effective hospital DP can be classified into four categories: policymakers', service providers' and recipient service behaviours; organization; payment and financing; and regulation (Gholizadeh, Janati, Delgoshaei, Gorji, & Tourani, 2018). DP is a multidisciplinary process for the continuity of care outside the hospital. The process includes identification, assessment, goal setting, planning, implementation, coordination and evaluation (Lin, Cheng, Shih, Chu, & Tjung, 2012).

A discharge planner advises, sets the process within a health institution, prepares and educates all staff and provides assistance in planning for discharge. The discharge planner plans, coordinates and communicates with patients, families and other healthcare providers while observing the DP process. A discharge planner can be a nurse, social worker, attending physician or case manager (Lin et al., 2012). DP involves (a) the early identification and assessment of patients needing DP assistance; (b) working with the patient, family and multidiscipline team to promote DP; (c) recommending alternatives for continued patient care and referring to accommodations, programs, or facilities to satisfy patient requirements and preferences; (d) collaboration with community organizations and care centres to encourage patients and address service gaps; and (e) support patients and families during hospital evaluation phases (Lin et al., 2012).

Numerous organizational, personal and socio‐cultural factors influence DP (Alreshidi, Long, & Cappleman, 2016) and the discharge planner must address the aspects that may affect patients during this transition period. First, physiologic factors include the assessment of patients' physical and functional abilities and their nutritional status and medications (LPN2009, 2006; Mehta, Nair, Rao, & Shukla, 2015). Second, psychological factors entail the assessment of patients' learning abilities and feelings about their diseases. Finally, discharge planners must assess social factors, such as the duration of care needed, the types of services available and the family involved in the care (LPN2009, 2006; Mehta et al., 2015).

The complexity of DP depends on the patient's needs. A simple DP should be applied when the patient does not need referrals outside the hospital. However, care planning requires assessment, preparation and effective communication with multidisciplinary teams. Simple DP may include check‐ups for smooth transport to home after ensuring readiness at home and a review of the patients' medication and nutritional needs (Goodman, Brompton, & Trust, 2010). The discharge planner must also verify whether patients and their families have received the specific information that they need. Complex DP should be introduced when patients' demands are more complicated and nurses are urgently needed to guarantee that patients have adequate support outside the hospital to achieve better health outcomes (An, 2014). If a patient needs a referral to an occupational or physiotherapist outside the hospital, or continuous nursing care at home, complex DP will need to be implemented (Goodman et al., 2010; Shimogai, Izawa, Kawada, & Kuriyama, 2019).

Due to the inconsistencies in the literature and the scarcity of consistent evidence regarding the assessment of nurses' knowledge, perception and practices of DP in acute care settings, the review questions for this study were as follows. (a) What are the main knowledge points of DP among nurses in acute care settings in the literature? (b) What does the literature say about nurses' attitudes towards DP in acute care settings? (c) What does the literature report about the DP practices of nurses in acute care settings?

## 3 STUDY DESIGN

We performed a systematic review using clearly formulated questions based on the background. All authors identified relevant studies, appraised their quality and summarized the evidence using an explicit methodology. We thoroughly reviewed studies that addressed nurses' knowledge, perception and practices of discharge planning in acute care settings.

## 4 METHODS

The researchers conducted an extensive nursing literature search using six key databases: CINAHL, MEDLINE, PubMed, Complete Academic search, Science Direct and Google Scholar. The search was limited to articles written in English and published in scientific journals, exclusively using human samples with a publication date in 1990 or later. The following keywords were used: discharge planning, nurses' perception, nurses' attitudes, nurses' knowledge and practices towards discharge planning. At the end of the search strategy, nine studies were found to address our literature review questions.

**Figure 1:**
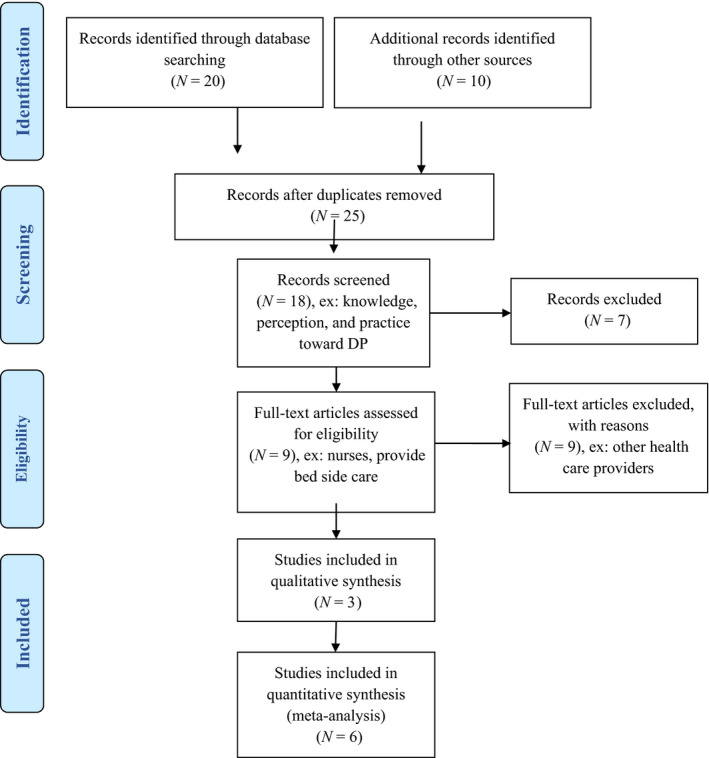
PRISMA flow diagram

The Preferred Reporting Items for Meta‐Analysis (PRISMA; Appendix S1) was used in this review, including the PRISMA checklist and flow chart. The PRISMA flow chart displayed in Figure 1 shows our comprehensive systematic search of the six databases, and the following keywords were used: (discharge planning) AND (acute care setting) AND ((knowledge) OR (attitude) OR (practice) OR (perception) OR (behavior)). The field was limited to “title/abstract,” and the publication type was limited to “journal article.” We performed reference tracking to identify additional, potentially relevant references. The aforementioned keywords and selection strategies yielded 30 articles. We removed duplicate articles and excluded seven additional articles after reviewing titles, abstracts and keywords. A total of 9 full‐text articles remained after excluding nine articles for reasons such as addressing DP among healthcare providers other than nurses. Figure 1 shows the nine selected articles.

Three researchers independently inspected the titles and abstracts to identify nurses' knowledge, perception and practices towards effective discharge planning (DP) in acute nursing units. The inclusion criteria were as follows: nurses' knowledge/skill, attitude/belief or practice/behaviour/implementation of DP; barriers or facilitators of effective/successful DP; original scientific studies; and written in English. The exclusion criteria were: reports or articles on DP from patients' standpoints or opinions; studies without clearly defined DP or among other health professionals/care providers; or systematic reviews, non‐research literature and conference presentations.

Three researchers independently assessed each study selected for retrieval for the quality of the methodology before inclusion in the review. The methodological quality assessment of the studies used in this study was supported with critical appraisal, using the Briggs Institute Meta‐Analysis of Statistics Assessment and Review Instrument (JBI‐MAStARI; Joanna Briggs Institute, 2014). With the JBI‐MAStARI tool, we followed the 3‐step search strategy: the initial limited search of selected databases and analysis of words in the titles and abstracts; the search using all identified keywords and index terms; and the reference list of all identified reports and articles searched for additional studies. Independent reviewers then assessed these articles for methodological validity before inclusion in the review.

## 5 ANALYSIS

Our data analysis/synthesis procedures were in line with the JBI‐MAStARI tool. The extracted data included specific details about the knowledge, perception and practices of DP from nurses' standpoint, meeting the review's specific objectives. Independent reviewers of this study read the included studies, extracted the results relevant to the review questions and compared the methodologies, samples, interventions and results. When there were discrepancies in the assessment or data extraction process, the researchers discussed and resolved the issues.

Comparative thematic analysis was used to develop broader pertinent themes for the current systematic review. The researchers read and assess the papers thoroughly to familiarize themselves with the subjects. Then, the authors made initial codes using the most common results from the reviewed papers to create themes. The two main themes were as follows: (a) demographic variables' role in implementing effective DP; and (b) nurses' knowledge, attitude and practices of effective DP in acute care settings. The researchers connected the yielded themes to the research questions to answer the review questions (Table 1).

**Table 1:** The selected nine articles of PRISMA systematic review

Study authors	Assessment for	Type of participants	Number of participants	Method	Main findings
Spataro(1994)	To examine the staff nurses' and nurses' managers' knowledge and sense of responsibility for DP	All RN nurses, And nurse manager, Full time or part time regardless years of experiences	178	Cross‐sectional, two questionnaires developed by Fritsch‐deBruyn and Cunningham (1990), to assess knowledge and sense of responsibility of nurses	On the knowledge test the mean score of the nurse managers' group was 14.23 (*SD* = 0.9701) out of a possible 15, which was higher than the staff nurse score of 13.81 (*SD* = 1.375) The nurse managers identified DP as a moderately high priority (score 7.29 out of a possible 10) relative to all other management duties
Mohammad et al. (2016)	To investigate nurses' knowledge towards DP for open‐heart patients	Nurses who are working in surgical word in cardiac hospitals and responsible for making discharge plan for open‐heart patients	A non‐probability (purposive sample), 52 nurses	Descriptive design, The questionnaire consisted of socio‐demographic characteristics Part, nurses knowledge about preparing DP for patient with open‐heart surgery and the last one consist of thirty questions, the answer was sure, unknown and not sure question answered by nurse and classify into this domains: 1. General information of nurses towards DP, Nurse knowledge related to medication 3. Nurse knowledge related to patients follow‐up, 4. Nurse knowledge related to patients activities 5. Nurse knowledge related to patients Nutrition 6. Nurse knowledge related to patients problems	Study shows level of nurses' knowledge towards DP for patient with open‐heart surgery were deficit in different domain, mainly in Nurses' knowledge towards patients follow‐up, and the highest level of nurses knowledge was nurses' knowledge towards patients activities
Lalani and Gulzar (2001)	To assess nurses’ knowledge, perceptions, and actual practice towards DP for patients	Nurse who are working in four medical‐surgical unit in Pakistan	15 nurses and 15 patients and15 files	Cross‐sectional design, knowledge and perception tool towards DP were used	Findings revealed that nurses lacked knowledge regarding DP which affects their practice in DP process; only 20% were able to clearly define what DP involved. about 67% of the nurses believed that it should be started on the day of discharge The nurses were spending only 2% of their total time from the 8‐hr shift with patients to prepare them for discharge
Chaboyer et al. (2002)	to explore perception of ICU nurses of their role of DP	ICU RN	65 RN	Cross‐sectional perception of DP tool, The scale came from Armitage S & Kavanagh K. Hospital nurses' perceptions of discharge planning for medical patients	results showed that most ICU nurses which is about 70% realize that DP is appropriate, useful, and should be taken as a priority in the ICU but about 40% stated that they lacked knowledge in the area of DP, also about half of participants believed that doctors provide patients sufficient information regarding DP, over one‐half believed that it should be a nurse responsibility. In addition, Majority of nurses found DP more paper works and time‐consuming process
Atwal (2002)	Nurses' perception towards DP	Nurses working on acute wards. The nurses who participated included nine from orthopaedics, six from acute medicine and four from elder care	19 nurses	Case study design with 19 nurses were interviewed, they also observe nurses and their communication with other healthcare providers in coordination for DP	Findings revealed that lack of time considered the major barrier for conducting and coordinating DP process
Chaboyer et al. (2004)	2004 to investigate the impact of a liaison nurse on ICU nurses' perception of discharge planning	ICU RN nurse	64 nurses within one Australian teaching hospital	Cross‐sectional design, nurses surveyed on tow time before and after implementing of the discharge liaison nurse,time 2 implementing after 10 months from initiation the role, authors used perception of Discharge planning scale	Results showed that self‐efficacy related to discharge planning did not changed over time, most of nurses still perceive discharge planning as a time‐consuming process Applying of discharge liaison nurse also has an effect on perception of nurses' towards DP, nurses' attitude become more positive towards DP, also they become more motivated in establishing and applying DP
Watts and Gardner (2005)	To explore how nurses perceived DP	Acute care settings	12 registered nurses	Qualitative, interview	one registered nurse believed that nurse should not be the coordinator of DP process, but the majority of nurses believed that their role is to communicate with other healthcare providers, nurses should be coordinate DP between multidisciplinary team, but this role was recognized as the main factor that could reinforce or disturb DP process, effective communication between nurse staff and medical staff enhance DP
Morris et al. (2012)	To explore perception around DP process	Acute care settings	461 registered nurse worked in full or part time in acute care hospital who responsible to discharge patients to home rather than ward	Modified version of the DP questionnaire developed by Chaboyer by changing the word of ICU to reflect the target population	Results showed that 76% of nurses believed that one of their role is to prepare patients for discharge and DP considered a significant process, but they believed that this process must be implemented by DP liaison team,also the results showed the some nurses about 21% lack understanding regarding this role
Chang et al. (2015)	To describe emergency department nurses' perception of factors that could affect the implementation of DP	ER nurses	25 RN	Qualitative via interview, factors that could affect the application of DP classified into three groups DP;1. As a neglected role to nurses in emergency department, 2.more workload, 3. the negative attitudes of patient and their families towards DP	Study revealed that hospital's culture which is not highly interested in DP, and the negative attitude of patients and their families as some nurses stated that they become frustrated when they deal with rude patients, or even if the patients not interested in DP, this theme was considered the major barrier to conduct DP.

## 6 RESULTS

The role of demographic variables in implementing effective DP was the first theme that this review yielded. One study examined factors affecting DP based on nurses' characteristics, including age, gender, marital status, work hours and education level (bachelor's degree or higher). It revealed a strong relationship between the level of education and the implementation of DP (*p*‐value .023). A higher level of education made nurses more systematic and logical in planning for discharge (Zakiyah & Basuki, 2017).

All the studies identified nurses as the cornerstone of DP in their settings. Only one study reported that DP was the responsibility of physicians and healthcare providers rather than nurses. All the studies focused on female nurses as the main part of their study populations. This focus underestimated the representation of male nurses. The participants' ages in the nine articles ranged from 18–53 years. Six articles listed addressing DP in only one setting as a major limitation in their studies (Atwal, 2002; Chaboyer, Foster, Kendall, & James, 2004; Chaboyer et al., 2002; Chang et al., 2015; Lalani & Gulzar, 2001; Morris, Winfield, & Young, 2012).

A review of the literature regarding demographic variables was conducted to determine their influence on nurses' knowledge, perception and practices of DP. In most studies, there were more females than males, making a comparison of gender‐based respondents difficult. Eleven men and 36 women were included in a study to examine the assessment of nurses' knowledge of DP (Mohammad, Fadil, & Ahmed, 2016) and 13 females and two male nurses in another (Lalani & Gulzar, 2001). Chaboyer and colleagues did not include gender‐based information because female respondents were predominant and male nurses were uncommon (Chaboyer et al., 2004; Chaboyer et al., 2002). A total of 124 females and 12 males were investigated by Morris et al. (2012), whereas 22 females and three males participated in another study (Chang et al., 2015).

One study reported strong evidence that the number of years of nursing experience affected patients' recovery and health outcomes positively (Atwal, 2002). In contrast, another study conducted in Iraq reported that new graduates and young nurses were more excited and interested in performing DP; this study was the only one to discover a relationship between age and DP (Mohammad et al., 2016).

The second theme that this review revealed was nurses' knowledge, attitude and practices towards effective DP in acute care settings. In terms of knowledge, one article showed that staff nurses perceived themselves as more qualified than other healthcare providers to perform DP (Spataro, 1994). Another article reported that nurses lacked knowledge in patients' follow‐up (Mohammad et al., 2016). One article showed that most nurses were unable to define DP and that lack of time was the source of the deficit in knowledge and application of DP (Lalani & Gulzar, 2001).

In terms of nurses' perception, three articles reported that nurses felt DP was a time‐consuming process, potentially affecting their engagement with it (Atwal, 2002; Chaboyer et al., 2004; Chaboyer et al., 2002). Chang et al. (2015) discovered the primary factor influencing nurses' DP implementation was an adverse attitude from patients and their relatives. One study showed that nurses believed that their role in DP was to communicate with other healthcare providers. Another showed that communication was considered the main reason for successful DP (Watts & Gardner, 2005). Hofmeyer and Clare (1999) acknowledged that hospital liaison nurses played a crucial role in the continuity of care of the elderly because they linked the communication between hospital and community nurses and general practitioners.

Likewise, Morris et al. (2012) found that nurses believed that the DP process should be implemented only by qualified liaison nurses; in turn, using a discharge liaison nurse affected nurses' perceptions of DP. Chaboyer et al. (2002) also found that doing so made nurses' attitudes more positive towards DP. They grew more motivated to establish and engage in DP themselves.

In this systematic review, numerous demographic and daily working experience factored into DP implementation among nurses in acute care settings. Although few studies have been conducted on nurses' knowledge, perceptions and practices of successful, effective discharge planning in acute care settings, this systematic review was able to highlight obstacles pertinent to these settings.

First, more attention needs to be paid to clarifying the study methodology to more precisely assess nurses' DP knowledge, attitudes and practices. Spataro (1994) examined staff nurses and nurse managers' DP knowledge and sense of responsibility in the United States with a convenience sample, yielding a survey of 102 participants with two questionnaires developed by Fritsch‐deBruyn and Cunningham in 1990. Part one of the questionnaire included eight multiple choices and seven true–false questions on general knowledge of DP. Part two had fifteen items on a Likert scale, ranging from 1–4 to gauge the nurses' perceived responsibility in DP. Part three was a demographic data questionnaire. The validity and reliability of the tool were not reported. Regarding the knowledge test, the mean score of the nurse managers' group was 14.23 (*SD* 0.9701), which was higher than that of the staff nurse, 13.81 (*SD* 1.375). However, this study showed that staff nurses perceived themselves as more capable of performing DP than their managers. The nurse managers identified DP as a moderately high priority (7.29 out of 10) among all of their management duties. The study cannot be generalized because it used only one institution.

Mohammad et al. (2016) conducted a study on nurses' knowledge of DP for open‐heart patients at three centres in Baghdad, Iraq. This study used a non‐probability (purposive) sample of 52 nurses working in the surgical wards of cardiac hospitals. They were responsible for creating discharge plans for open‐heart patients. The questionnaire consisted of three parts. The first part concerned socio‐demographic characteristics. The second part covered nurses' knowledge of preparing DP for patients with open‐heart surgery. The last part consisted of thirty questions, to which the responding nurses could answer "sure," "unknown" or "not sure." The questions were classified as nurses' knowledge of: (a) DP in general; (b) medication; (c) patients' follow‐up; (d) patients' activities; (f) patients' nutrition; and (g) patients' problems. The nurses' knowledge level of DP was low in various domains, especially concerning patient follow‐up. The highest level of knowledge occurred in the domain of patient activities. The reliability of the tool was 0.75. The limitation of this study was the small sample size. In addition, female nurses were more common in the three centres.

A study conducted by Lalani and Gulzar (2001) in Pakistan to assess nurses' knowledge, perceptions and actual practice of DP used a cross‐sectional design and two semi‐structured questionnaires for patients and nurses. Convenience samples of 15 nurses and 15 patients were gathered from four medical‐surgical units. The fifteen patient files were also studied. The study revealed that the nurses lacked DP knowledge, which affected their process. Only 20% were able to clearly define DP and what the process entails. Of the nurses, 67% believed that DP should begin on the day of discharge. This study was considered weak because of the small convenience sample size, which affected the generalization of the results.

Chaboyer et al. (2002) studied intensive care unit (ICU) nurses' perceptions of their role in DP, surveying a convenience sample of 65 Registered Nurses from one ICU. Of the nurses, 70% realized that DP should be considered an appropriate, useful priority in the ICU, whereas about 40% stated that they lacked knowledge of DP. About half of the participants believed that doctors provided patients with sufficient discharge information, whereas half believed that it should be a nurse's responsibility. The authors used a reliable, valid tool to gauge DP perceptions with a Cronbach's alpha coefficient of 0.73 for the 14 items.

In another study, Chaboyer et al. (2004) investigated the impact of a liaison nurse on ICU nurses' perceptions of discharge planning. A sample of 64 nurses from one Australian teaching hospital was surveyed at two intervals. Before and after implementing the discharge liaison, reassessment was performed ten months after the course was initiated. The authors used the Perception of the Discharge Planning scale, which applies a 4‐point scale ranging from not at all true (1) to exactly true (4). The internal consistency was 0.90. Construct validity was measured using a single, unidimensional construct of self‐efficacy. Their results showed that the self‐efficacy of DP was consistent over time; most nurses continued to perceive DP as a time‐consuming process. However, the ICU nurses became more positive towards discharge planning, felt they took on a greater role in establishing and applying it and reported a greater understanding of it. Limitations of the study included the use of only one site and a small sample size. The internal validity of the study design was vulnerable to threats because of maturation because of the limited timeframe over which outcomes were measured.

Another study (Atwal, 2002) was conducted in a British teaching hospital regarding nurses' perception of DP in an acute care setting. Atwal interviewed and observed 19 nurses while working in the unit. The nurses communicated with other healthcare providers in preparation for DP. A lack of time was considered the major barrier for conducting and coordinating the DP process. One of the limitations of this study was the use of only one healthcare setting.

In a qualitative study conducted in a public hospital to explore how nurses perceived DP in an acute care setting, Watts and Gardner (2005) interviewed 12 registered nurses. Only one registered nurse believed that nurses should not be the coordinator of the DP process. Most nurses believed that their role was to communicate with other healthcare providers. The study also reported that nurses should coordinate DP among multidisciplinary personnel. This role was recognized as the main factor for strengthening or disturbing the DP process because of the necessity of collaboration and communication between nurses and medical staff. The inability to generalize the results of the study was one of the limitations (Watts & Gardner, 2005).

Morris et al. (2012) conducted a study with a convenience sample of 461 registered nurses' working full time or part time, who were responsible for discharging patients to home rather than the ward. The researchers used a modified version of the DP questionnaire developed by Chaboyer by changing the wording from that of an ICU ward to reflect the target population. Its reliability was 0.70. The questionnaire contained Likert‐style statements with five answers (strongly disagree, disagree, unsure, agree, strongly agree). Of the nurses, 76% believed that one of their roles was to prepare patients for discharge and that DP was a significant process. However, they also believed that the DP liaison team must implement the process. Their results showed that about 21% of nurses lacked understanding of DP. One of the limitations was that the study was conducted in one hospital.

In a recent qualitative study, Chang et al. (2015) investigated nurses' perceptions of factors affecting DP implementation in the emergency department. The researchers surveyed 25 nurses and found three categories of factors. The first was the neglected role of nurses in the emergency department. The second was the heavy workload. The third was the negative attitudes of patients and their families towards DP. This study revealed that hospital culture did not reflect a strong interest in DP and the negative attitude of patients and their families presented an obstacle in conducting DP. Some nurses stated that they became frustrated while dealing with rude patients and that patients were not interested in DP. The results are not generalizable to the broader population of emergency nurses.

Published studies in nurses' practices of DP are limited. Although nurses showed an awareness level of DP, the literature showed that only 2% of an 8‐hr shift was spent preparing patients for discharge. Staff nurses can perform DP and assess patients' learning abilities effectively (Spataro, 1994). However, patients' records revealed that nursing assessments were incomplete and the application of DP was fragmented (Lalani & Gulzar, 2001). About 80% of nurses believed that DP should be started when patients were admitted to the hospital (Morris et al., 2012).

Despite the significance of DP, only nine studies were found in a literature review assessing nurses' knowledge, perceptions and practices of DP. The findings were disorganized in the literature. No published studies were conducted in Middle Eastern countries, except Iraq. Most studies focused on nurses' perceptions rather than their knowledge and practices. The scarcity of research raises the importance of conducting such a study in Jordan.

Numerous factors are involved in the implementation of successful and effective DP. Issues that should be addressed include nurses' low knowledge levels of patients' activities and discharge, inability to define DP, debates over DP timing and preparation, facing negative attitudes from patients and family members negative and considering DP as time‐consuming paperwork that the physician should do. There is an urgent need to combat these obstacles to implementing successful discharge planning. Better time management skills during work and relying on liaison nurses may improve DP in acute care settings.

## CONFLICT OF INTEREST

All authors declare that they have no conflict of interest.

## AUTHOR CONTRIBUTIONS

We hereby confirm that all listed authors meet the authorship criteria and that all authors are in agreement with the content of the manuscript. AH, IH: Study conception and design. AH, MA: Data collection and analysis. AH, IH, MA: Data interpretation. AH, IH, MA: Manuscript preparation. AH, IH, MA: Final approval of the manuscript version to be published.

## Supporting information

Appendix S1

## References

[nop2547-bib-0020] Abdulfattah, D. , & Mushcab, H. (2017). Nurse‐led Discharge in Saudi Arabia: A Thematic Investigation of the Literature. EC Nutrition, 9(4), 188–195.

[nop2547-bib-0001] Alreshidi, N. , Long, T. , & Cappleman, J. (2016). Factors influencing discharge planning in neonatal intensive care units in Saudi Arabia: A systematic review. Gulf Medical Journal, 5(1), 27–35.

[nop2547-bib-0002] An, D. (2014). Cochrane review brief: Discharge planning from hospital to home. The Online Journal of Issues in Nursing, 20(2). https://ojin.nursingworld.org/MainMenuCategories/ANAMarketplace/ANAPeriodicals/OJIN/Columns/Cochrane‐Review‐Briefs/Discharge‐Planning.html 26882432

[nop2547-bib-0003] Atwal, A. (2002). Nurses' perceptions of discharge planning in acute health care: A case study in one British teaching hospital. Journal of Advanced Nursing, 39(5), 450–458. 10.1046/j.1365-2648.2002.02310.x 12175354

[nop2547-bib-0004] Birjandi, A. , & Bragg, L. (2008). Discharge planning handbook for healthcare: Top 10 secrets to unlocking a new revenue pipeline. New York, NY: Taylor & Francis: CRC group.

[nop2547-bib-0005] Chaboyer, W. , Foster, M. , Kendall, E. , & James, H. (2004). The impact of a liaison nurse on ICU nurses' perceptions of discharge planning. Australian Critical Care, 17(1), 25–32. 15011994 10.1016/s1036-7314(05)80047-5

[nop2547-bib-0006] Chaboyer, W. , Foster, M. , Kendall, E. , James, H. , Chaboyer, W. , Kendall, E. , & James, H. (2002). ICU nurses’ perceptions of discharge planning: A preliminary study. Intensive and Critical Care Nursing, 3397(02), 90–95. 10.1016/S0964-3397(02)00022-8 12353656

[nop2547-bib-0007] Chang, W. , Goopy, S. , Lin, C. C. , Barnard, A. , Liu, H. E. , & Han, C. Y. (2015). Registered nurses and discharge planning in a Taiwanese ED: A neglected issue*?* Clinical Nursing Research, 25(5), 512–531. 10.1177/1054773815584138 25940582

[nop2547-bib-0009] Flink, M. , & Ekstedt, M. (2017). Planning for the discharge, not for patient self‐management at home – An observational and interview study of hospital discharge. International Journal of Integrated Care, 17(6), 1–10. 10.5334/ijic.3003 PMC5854016 29588634

[nop2547-bib-0010] Fritsch‐deBruyn, R. , & Cunningham, H. (1990). Acheck on nurses' knowledge and sense of responsibility for discharge planning. Journal of Nursing Staff Development, 6(4), 173–176, 185. 2380771

[nop2547-bib-0011] Gholizadeh, M. , Janati, A. , Delgoshaei, B. , Gorji, H. A. , & Tourani, S. (2018). Implementation requirements for patient discharge planning in health system: A qualitative study in Iran. Ethiopian Journal of Health Sciences, 28(2), 157–168. 10.4314/ejhs.v28i2.7 29983513 PMC6016349

[nop2547-bib-0012] Goodman, H. , Brompton, R. , & Trust, F. (2010). Discharge from hospital: The importance of planning. British Journal of Cardiac Nursing, 5(6), 274–280.

[nop2547-bib-0013] Hofmeyer, A. , & Clare, J. (1999). The role of the hospital liaison nurse in effective discharge planning for older people: Perspectives of discharge planners. Contemporary Nurse, 8(3), 99–106. 10.5172/conu.1999.8.3.99 11132006

[nop2547-bib-0014] Joanna Briggs Institute (2014). Joanna Briggs Institute reviewers’ manual: 2014 edition. Adelaide, SA: The Joanna Briggs Institute.

[nop2547-bib-0015] Lalani, N. S. , & Gulzar, A. Z. (2001). Nurses' role in patients'discharge planning at the Aga Khan University Hospital, Pakistan. Journal for Nurses in Professional Development, 17(6), 314–319. 11840014

[nop2547-bib-0016] Lin, C. , Cheng, S. , Shih, S. , Chu, C. , & Tjung, J. (2012). Discharge planning q. International Journal of Gerontology, 6(4), 237–240. 10.1016/j.ijge.2012.05.001

[nop2547-bib-0008] LPN2009 . (2006). Charting Checkup: Planning your patient's discharge, (Vol. 2(2), pp. 10–12). Wolters Kluwer: Lippincott Williams & Wilkins (LWW).

[nop2547-bib-0017] Mehta, S. , Nair, J. , Rao, S. , & Shukla, K. (2015). Role of discharge planning and other determinants in total discharge time at a large tertiary care hospital. CHRISMED Journal of Health Research, 2, 46–50. 10.4103/2348-3334.149345

[nop2547-bib-0018] Mohammad, M. , Fadil, A. , & Ahmed, S. A. (2016). Assessment of nurses’ knowledge concerning discharge planning for patients ’ with open heart surgery in Cardiac Centre at Baghdad. City, 6(10), 162–167.

[nop2547-bib-0019] Morris, J. , Winfield, L. , & Young, K. (2012). Registered nurses’ perceptions of the discharge planning process for adult patients in an acute hospital. Journal of Nursing Education and Practice, 2(1), 28–38. 10.5430/jnep.v2n1p28

[nop2547-bib-0021] Nordmark, S. , Zingmark, K. , & Lindberg, I. (2016). Process evaluation of discharge planning implementation in healthcare using normalization process theory. BMC Medical Informatics and Decision Making, 16, 48 10.1186/s12911-016-0285-4 27121500 PMC4847180

[nop2547-bib-0022] Pellett, C. (2016). Discharge planning: Best practice in transitions of care. British Journal of Community Nursing, 21(11), 542–548. 27809581 10.12968/bjcn.2016.21.11.542

[nop2547-bib-0023] Shimogai, T. , Izawa, K. P. , Kawada, M. , & Kuriyama, A. (2019). Factors affecting discharge to home of medical patients treated in an intensive care unit. International Journal of Environmental Research and Public Health, 16(22), 4324 10.3390/ijerph16224324 PMC6887772 31698814

[nop2547-bib-0025] Spataro, J. A. (1994). An investigation of staff nurses' and nurse managers' knowledge and sense of responsibility for discharge planning. Doctoral dissertation, Bellarmine College.

[nop2547-bib-0026] Watts, R. , & Gardner, H. (2005). Nurses’ perceptions of discharge planning. Nursing & Health Sciences, 7(3), 175–183. 10.1111/j.1442-2018.2005.00229.x 16083480

[nop2547-bib-0027] Zakiyah, A. , & Basuki, D. (2017). Relationship between nurse characteristics with discharge planning implementation. International Journal of Nursing and Midwifery Science (Ijnms), 1(2), 193–197.

